# A new species of the freshwater crab genus *Potamonemus* Cumberlidge & Clark, 1992 (Crustacea, Potamonautidae) endemic to the forested highlands of southwestern Cameroon, Central Africa

**DOI:** 10.3897/zookeys.1017.60990

**Published:** 2021-02-15

**Authors:** Pierre A. Mvogo Ndongo, Thomas von Rintelen, Neil Cumberlidge

**Affiliations:** 1 Département de Gestion des Écosystèmes Aquatiques, Institut des Sciences Halieutiques, Université de Douala à Yabassi, PO. Box. 7236, Douala-Bassa, Cameroun Museum für Naturkunde Berlin Germany; 2 Museum für Naturkunde, Leibniz Institute for Evolution and Biodiversity Science, Invalidenstraße 43, 10115, Berlin, Germany Université de Douala à Yabassi Douala-Bassa Cameroon; 3 Department of Biology, Northern Michigan University, Marquette, MI, 49855-5376, USA Northern Michigan University Marquette United States of America

**Keywords:** Afrotropical region, conservation, identification key, mtDNA, taxonomy

## Abstract

A new species of freshwater crab of the genus *Potamonemus* Cumberlidge & Clark, 1992 is described from Mount Manengouba Reserve and Bakossi National Park in the tropical rainforests of southwestern Cameroon, Central Africa. *Potamonemusman***sp. nov.** is recognized by characters of the carapace and chelipeds. In addition, a phylogenetic analysis based on partial sequences of three mitochondrial DNA genes (COI, 12S rRNA, and 16S rRNA) that included representatives of all other freshwater crab genera found in Cameroon recovered each of the new species as a distinct lineage. A diagnosis and illustrations of the new species are provided, and it is compared to the other species of *Potamonemus*. Brief notes are provided on the ecology of the new species and the two other species of *Potamonemus*. An identification key to the species of *Potamonemus* is provided. The conservation status of the genus is discussed.

## Introduction

*Potamonemus* Cumberlidge & Clark, 1992 is one of five genera of freshwater crabs currently known from Cameroon. The other four species are *Buea* Cumberlidge, Mvogo Ndongo, Clark & Daniels, 2019, *Louisea* Cumberlidge, 1994, *Potamonautes* MacLeay, 1838, and *Sudanonautes* Bott, 1955 (Cumberlidge 1987, 1989, 1993a, b, c, 1994a, b; 1999; Cumberlidge and Boyko 2001; Cumberlidge and Clark 1992; Cumberlidge et al. 2019; Mvogo Ndongo et al. 2017a, b, c, 2018, 2019, 2020). *Potamonemus* was originally established as a monotypic genus for *P.mambilorum* Cumberlidge & Clark, 1992, and in the following year two more species were described, namely *P.asylos* Cumberlidge, 1993 and *P.sachsi* Cumberlidge, 1993. Subsequent phylogenetic analyses of the Afrotropical freshwater crab fauna (Daniels et al. 2006, 2015) supported the close relationship between *P.mambilorum* and *P.sachsi* but indicated that *P.asylos* formed a separate genetic lineage from these two species. Recently, *P.asylos* was assigned to a new genus, *Buea* Cumberlidge, Mvogo Ndongo, Clark & Daniels, 2019. Mvogo Ndongo et al. (2020) recently described three additional *Buea* species, including *B.bangem* Mvogo Ndongo, von Rintelen, Tomedi-Tabi & Cumberlidge, 2020, *B.mundemba* Mvogo Ndongo, von Rintelen & Cumberlidge in Mvogo Ndongo, von Rintelen, Tomedi-Tabi and Cumberlidge 2020, and *B.nlonako* Mvogo Ndongo, von Rintelen & Cumberlidge in Mvogo Ndongo, von Rintelen, Tomedi-Tabi and Cumberlidge 2020.

Cumberlidge et al. (2019) and Mvogo Ndongo et al. (2020) established that *Buea* is endemic to southwestern Cameroon and that *Potamonemus* is a more widely distributed genus, with a range including southwestern Cameroon and eastern Nigeria. Cumberlidge et al. (2019) revised the diagnostic characters of *Potamonemus* as a 2-segmented mandibular palp lacking a lobe or anterior flap at the junction between the two segments, a G1 with a slim, outward-curving and elongated TA (TA/SS 0.63) that lacks marginal setae and tapers to a pointed tip, and a G2 with a remarkably short TA (TA/SS 0.13). The three protected areas surveyed in the present study for freshwater decapods are located in a region of southwestern Cameroon which has been recognised as a biodiversity hotspot for several other freshwater taxa.

Extensive systematic surveys carried out from 2017 to 2020 in the lowland and upland zones of the tropical rainforests of southwestern Cameroon resulted in the collection of several new taxa, including a new species of *Potamonemus*. The purpose of the present study is to describe this new species using an integrative approach based on morphological characters and molecular genetic data and to provide a key to the three species now assigned to this genus. The molecular analysis based on three partial mitochondrial genes (COI, 12S rRNA, and 16S rRNA) also recovers the three species as separate genetic lineages within *Potamonemus*. For all the species treated in this study we also provide notes on their ecology and conservation.

## Materials and methods

### Sampling

Field surveys of freshwater decapods were conducted in 2017 at Mount Manengouba Reserve and Bakossi National Park, and in 2018 and 2020 at Nlonako Ecological Reserve. Survey transects were made in each study area. Crabs were collected from small rivers using nylon fishnets and dip nets, and near small permanent streams where crabs were found in puddles, under fallen leaves, under stones, and in burrows. The amount of disturbance of the habitat and the various threats to freshwater organisms, including to freshwater crabs, were evaluated during structured discussions with local people.

### Morphological analyses

All measurements (in mm) were taken with digital callipers. The terminology used follows Cumberlidge (1999), and the classification follows Ng et al. (2008). Characters of the gonopods, carapace, thoracic sternum, chelipeds, third maxillipeds, and mandibles were examined in detail, and photographs were taken using a Leica microscope (model Z16A POA), LAS V4, and Helicon Focus 6.7.1 software. Post processing of the images was undertaken using Adobe Photoshop CC5 and Photo Impact. The newly collected specimens were deposited in the Museum für Naturkunde, Berlin, Germany (**ZMB**). Other material is deposited in the Institute of Fisheries and Aquatic Sciences, University of Douala at Yabassi (**IFAS**).

### Abbreviations used

**A** pleonal (abdominal) segment or pleomere;

**A5/A6** sulci between adjacent pleomeres;

**a.s.l.** above sea level;

**CW** carapace width measured at widest point;

**CL** carapace length measured along medial line from anterior to posterior margin;

**CH** carapace height measured at maximum height of cephalothorax;

**E** episternite;

**FW** front width measured along anterior frontal margin between inner angles of orbits;

**G1** male first gonopod;

**G2** male second gonopod;

**P2–5** pereiopods 2–5 or ambulatory legs 1–4;

**SS** subterminal segment of G1 or G2;

**S4/E4** (S4/E4, S5/E5, S6/E6, S7/E7) episternal sulci between adjacent thoracic sternites and episternites;

**S** thoracic sternite;

**S1/S2** (or S2/S3, S4/S5, S5/S6, S6/S7) sternal sulci between adjacent thoracic sternites;

**TA** terminal article of G1 or G2;

**TS** terminal segment of mandibular palp.

Details for DNA extraction, DNA sequencing, PCR, and molecular phylogenetic analyses are given by Mvogo Ndongo et al. (2019, 2020). All sequences used in this study are given in Table 1.

## Systematic account

### Infraorder Brachyura Latreille, 1802

#### Superfamily Potamoidea Ortmann, 1896

**Family Potamonautidae Bott, 1970**

**Subfamily Potamonautinae Bott, 1970**

##### Potamonemus man sp. nov.

Taxon classificationAnimaliaDecapodaPotamonautidae

DD1BB93D-70B7-5758-AA2B-FF9FE096C762

http://zoobank.org/58FD0C15-4CB9-4561-8453-B98255BBEE25

**Figure 1.**
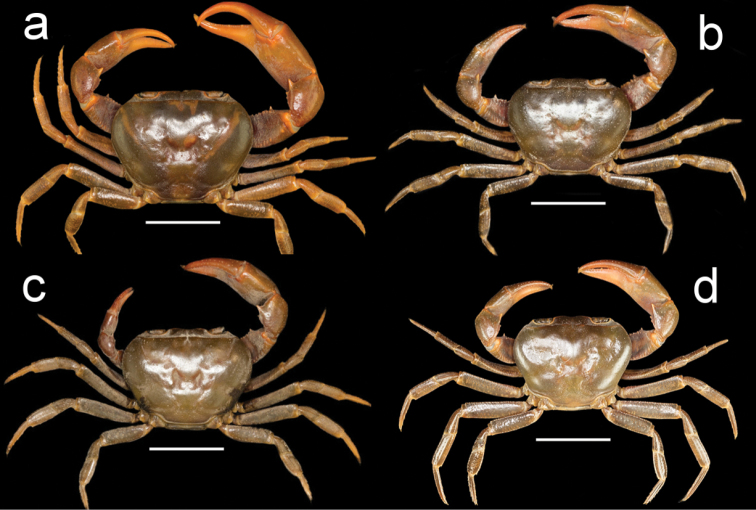
Species of *Potamonemus* from southwestern Cameroon, whole animal, dorsal view **a** largest adult male (CW 29 mm) of *P.mambilorum* from Mount Nlonako (ZMB Crust. 32428) **b** adult male (CW 28 mm) of *P.mambilorum* from small stream on Mount Manengouba (ZMB Crust. 30326) **c** adult male (CW 31 mm) of *P.man* sp. nov. from Bakossi National Park (ZMB Crust. 30328) **d** adult male, holotype (CW 25 mm) of *P.man* sp. nov. from Man Lake, Mount Manengouba (ZMB Crust. 30320). Scale bars: 15 mm (**a**), 16 mm (**b**), 17 mm (**c**), 16 mm (**d**).

**Figure 2.**
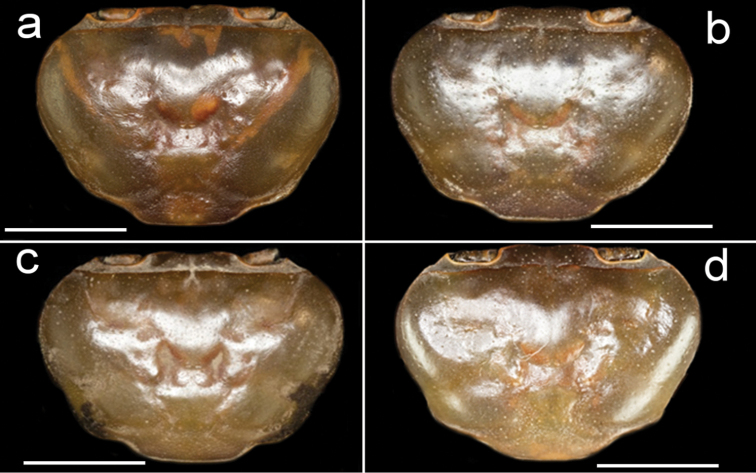
Species of *Potamonemus* from southwestern Cameroon, dorsal view **a** largest adult male (CW 29 mm) of *P.mambilorum* from Mount Nlonako (ZMB Crust. 32428) **b** adult male (CW 28 mm) of *P.mambilorum* from small stream on Mount Manengouba (ZMB Crust. 30326) **c** adult male (CW 31 mm) of *P.man* sp. nov. from Bakossi National Park (ZMB Crust. 30328) **d** adult male, holotype (CW 25 mm) of *P.man* sp. nov. from Man Lake, Mount Manengouba (ZMB Crust. 30320). Scale bars: 13 mm (**a**), 13 mm (**b**), 14 mm (**c**), 11 mm (**d**).

**Figure 3.**
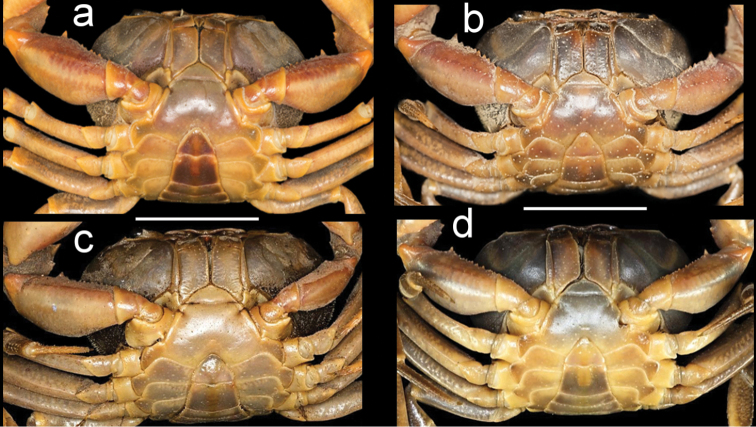
Species of *Potamonemus* from southwestern Cameroon, ventral view **a** largest adult male (CW 29 mm) of *P.mambilorum* from Mount Nlonako (ZMB Crust. 32428) **b** adult male, holotype (CW 28 mm) of *P.mambilorum* from small stream on Mount Manengouba (ZMB Crust. 30326) **c** adult male (CW 31 mm) of *P.man* sp. nov. from Bakossi National Park (ZMB Crust. 30328) **d** adult male, holotype (CW 25 mm) of *P.man* sp. nov. from Man Lake, Mount Manengouba (ZMB Crust. 30320). Scale bars: 16 mm (**a**), 17 mm (**b**), 16 mm (**c**), 17 mm (**d**).

**Figure 4.**
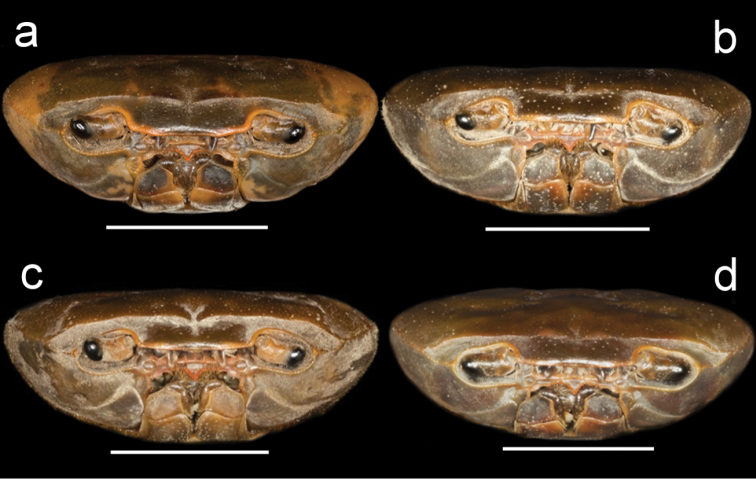
Species of *Potamonemus* from southwestern Cameroon, frontal view **a** largest adult male (CW 29 mm) of *P.mambilorum* from Mount Nlonako (ZMB Crust. 32428) **b** adult male, holotype (CW 28 mm) of *P.mambilorum* from small stream on Mount Manengouba (ZMB Crust. 30326) **c** adult male (CW 31 mm) of *P.man* sp. nov. from Bakossi National Park (ZMB Crust. 30328) **d** adult male, holotype (CW 25 mm) of *P.man* sp. nov. from Man Lake, Mount Manengouba (ZMB Crust. 30320). Scale bars: 13 mm (**a**), 12 mm (**b**), 14 mm (**c**), 11 mm (**d**).

**Figure 5.**
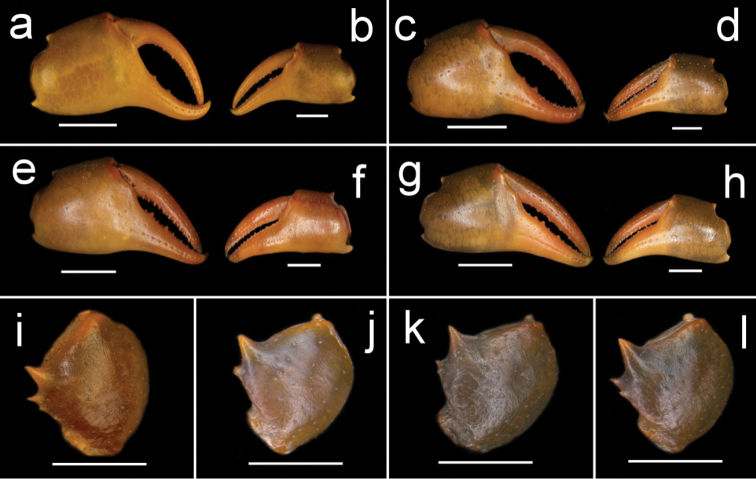
Frontal view of the right and left chelae of adult males of species of *Potamonemus* from southwestern Cameroon **a, b** largest adult male (CW 29 mm) of *P.mambilorum* from Mount Nlonako (ZMB Crust. 32428) **c, d** adult male (CW 28 mm) of *P.mambilorum* from small stream on Mount Manengouba (ZMB Crust. 30326) **e, f** largest adult male, holotype (CW 31 mm) of *P.man* sp. nov. from Bakossi National Park (ZMB Crust. 30328) **g, h** adult male, holotype (CW 25 mm) of *P.man* sp. nov. from Man Lake, Mount Manengouba (ZMB Crust. 30320). Dorsal view of the right cheliped carpus of adult males of species of *Potamonemus***i**, largest adult male (CW 29 mm) of *P.mambilorum* from Mount Nlonako (ZMB Crust. 32428) **j** adult male (CW 28 mm) of *P.mambilorum* from small stream on Mount Manengouba (ZMB Crust. 30326) **k** adult male (CW 31 mm) of *P.man* sp. nov. from Bakossi National Park (ZMB Crust. 30328) **l** adult male, holotype (CW 25 mm) of *P.man* sp. nov. from Man Lake, Mount Manengouba (ZMB Crust. 30320). Scale bars: 5 mm (**a, c, g**), 2.5 mm (**b, d, f, h**), 5 mm (**i, j, k, l**).

**Figure 6.**
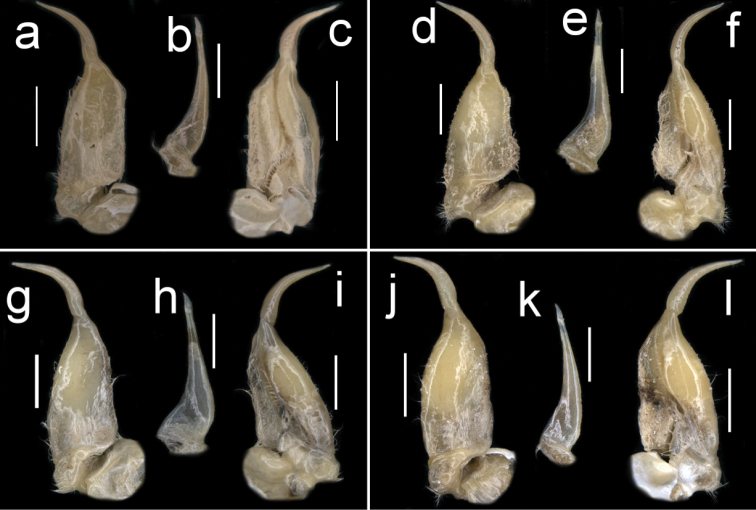
Dorsal view of left G1 (**a, d, g, j**) and ventral view of left G1 (**c, f, i, l**) of adult males of species of *Potamonemus* from southwestern Cameroon. **a, c** largest adult male (CW 29 mm) of *P.mambilorum* from Mount Nlonako (ZMB Crust. 32428) **d, f** adult male (CW 28 mm) of *P.mambilorum* from small stream on Mount Manengouba (ZMB Crust. 30326) **g, i** adult male (CW 31 mm) of *P.man* sp. nov. from Bakossi National Park (ZMB Crust. 30328) **j, l** adult male, holotype (CW 25 mm) of *P.man* sp. nov. from Man Lake, Mount Manengouba (ZMB Crust. 30320). Ventral view of G2 of adult males of species of *Potamonemus* from southwestern Cameroon **b** largest adult male (CW 29 mm) of *P.mambilorum* from Mount Nlonako (ZMB Crust. 32428) **e** adult male (CW 28 mm) of *P.mambilorum* from small stream on Mount Manengouba (ZMB Crust. 30326) **h** adult male (CW 31 mm) of *P.man* sp. nov. from Bakossi National Park (ZMB Crust. 30328) **k** adult male, holotype (CW 25 mm) of *P.man* sp. nov. from Man Lake, Mount Manengouba (ZMB Crust. 30320). Scale bars: 2 mm (**a–k**).

**Figure 7.**
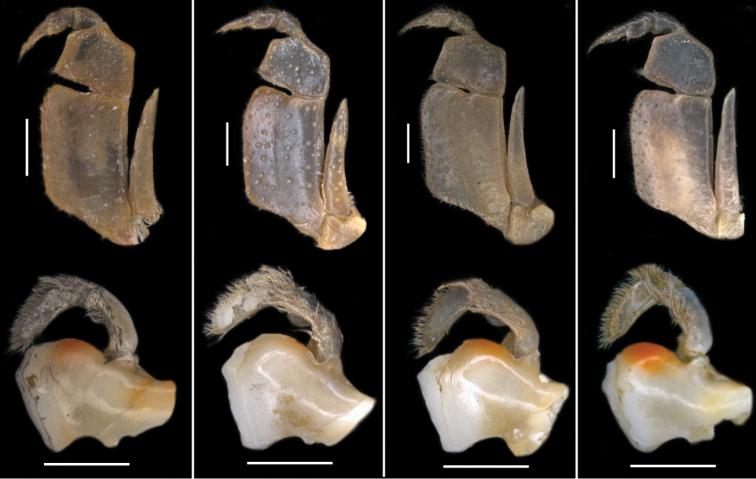
Frontal view of the left third maxilliped of adult males of species of *Potamonemus* from southwestern Cameroon **a** largest adult male (CW 29 mm) of *P.mambilorum* from Mount Nlonako (ZMB Crust. 32428) **b** adult male (CW 28 mm) of *P.mambilorum* from small stream on Mount Manengouba (ZMB Crust. 30326) **c** adult male (CW 31 mm) of *P.man* sp. nov. from Bakossi National Park (ZMB Crust. 30328) **d** adult male, holotype (CW 25 mm) of *P.man* sp. nov. from Man Lake, Mount Manengouba (ZMB Crust. 30320). Frontal view of the left mandible of adult males of species of *Potamonemus* from southwestern Cameroon **e** largest adult male (CW 29 mm) of *P.mambilorum* from Mount Nlonako (ZMB Crust. 32428) **f** adult male (CW 28 mm) of *P.mambilorum* from small stream on Mount Manengouba (ZMB Crust. 30326) **g** adult male (CW 31 mm) of *P.man* sp. nov. from Bakossi National Park (ZMB Crust. 30328) **h** adult male, holotype (CW 25 mm) of *P.man* sp. nov. from Man Lake, Mount Manengouba (ZMB Crust. 30320). Scale bars: 5 mm (**a–d**), 2 mm (**e–h**).

Figures 1d , 2d , 3d , 4d , 5g, h, l , 6j–l , 7d, h Common name: Man Lake freshwater crab

###### Holotype.

Adult ♂ (CW 24.51 mm, CL 17.09 mm, CH 9.62 mm, FW 7.62 mm), Cameroon, Southwest Region, Mount Manengouba Ecological Reserve, Man Lake, Mount Manengouba (5.02414, 9.82142), 1,958 m a.s.l., 14 March 2017, coll. P.A. Mvogo Ndongo (ZMB Crust. 30320).

###### Paratypes.

1 adult ♂ (CW 21.37 mm, CL 15.61 mm, CH 8.45 mm, FW 6.69 mm), 1 adult ♀ (CW 23.55 mm, CL 16.87 mm, CH 10.09 mm, FW 7.60 mm), Cameroon, Southwest Region, Mount Manengouba Ecological Reserve, Man Lake, Mount Manengouba (5.03604, 9.82906), 1,958 m a.s.l., 14 March 2017, coll. P.A. Mvogo Ndongo (ZMB Crust. 30324). 2 adult ♂ (CW 20.12 mm, CL 14.64 mm, CH 7.92 mm, FW 6.76 mm; CW 20.40 mm, CL 14.73 mm, CH 8.16 mm, FW 6.63 mm); 3 subadult ♂ (CW 18.46 mm, CL 13.89 mm, CH 7.40 mm, FW 6.16 mm; CW 19.38 mm, CL 13.78 mm, CH 7.78 mm, FW 6.63 mm; CW 14.05 mm, CL 10.67 mm, CH 5.84 mm, FW 5.30 mm), Cameroon, Southwest Region, Mount Manengouba Ecological Reserve, Man Lake, Mount Manengouba (5.03604, 9.82906), 1,958 m a.s.l., 14 March 2017, coll. P.A. Mvogo Ndongo (IFAS-017); 4 adult ♀ (CW 19.39 mm, CL 14.07 mm, CH 7.74 mm, FW 6.48 mm; CW 17.37 mm, CL 12.46 mm, CH 6.94 mm, FW 6.33 mm; CW 16.88 mm, CL 12.06 mm, CH 6.36 mm, FW 5.20 mm), Cameroon, Mount Manengouba Ecological Reserve, Man Lake, Mount Manengouba (5.03604, 9.82906), 1,958 m a.s.l., 14 March 2017, coll. P.A. Mvogo Ndongo (IFAS-018).

###### Other material.

Bakossi National Park (Figs 1c, 2c, 3c, 4c, 5e, f, j, 6g–i, 7c, g). 1 adult ♂ (CW 30.41 mm, CL 20.57 mm, CH 12.50 mm, FW 9.32 mm), 1 adult ♀ (CW 27.48 mm, CL 20.06 mm, CH 11.31 mm, FW 8.19 mm), Cameroon, Southwest Region, Bakossi National Park (5.031083, 9.687528), 1,253 m a.s.l., 15 March 2017, coll. P. A. Mvogo Ndongo (ZMB Crust. 30328). 5 adult ♂ (CW 27.61 mm, CL 19.37mm, CH 11.39 mm, FW 8.65 mm; CW 26.54 mm, CL 19.09 mm, CH 10.80 mm, FW 9.13 mm; CW 25.55 mm, CL 18.68 mm, CH 10.58 mm, FW 8.41 mm; CW 25.3 mm, CL 18.05 mm, CH 10.70, FW 8.41 mm) (IFAS-014); 2 subadult ♂ (CW 22.17 mm, CL 15.76 mm, CH 9.04 mm, FW 7.35 mm; CW 22.02 mm, CL 15.67 mm, CH 9.06 mm, FW 7.49 mm; CW 22.04 mm, CL 16.00 mm, CH 9.22 mm, FW 7.65 mm; CW 21.35 mm, CL 15.19 mm, CH 8.76 mm, FW 6.81 mm), Cameroon, Southwest Region, Bakossi National Park (5.031083, 9.687528), 1,248 m a.s.l., 15 March 2017, coll. P.A. Mvogo Ndongo (IFAS-015). 2 subadult ♀ (CW 23.76 mm, CL 17.57 mm, CH 9.88 mm, FW 7.68 mm; CW 23.31 mm, CL 16.63 mm, CH 9.08 mm, FW 7.59 mm), Cameroon, Southwest Region, Bakossi National Park (05.031083, 9.687528), 1,248 m a.s.l., 15 March 2017, coll. P.A. Mvogo Ndongo (IFAS-016).

**Table 1.** Species and specimens of *Louisea*, *Buea*, *Potamonemus*, *Sudanonautes*, and *Potamonautes* and the outgroup taxa included in the molecular analysis. All measurements in mm.

Species	Locality	Museum number	Reference study	GenBank accession number		
				CO1	12S rRNA	16S rRNA
*Louiseankongsamba* (CW 20.0)	Mt. Nlonako	ZMB Crust. 31618	Mvogo Ndongo et al. 2019	MN188072	MN217386	MN217393
*Louiseabalssi* (CW 14.8)	Manengouba	ZMB Crust.29628	Mvogo Ndongo et al. 2019	MN188070	MN217384	MN217391
*Louiseaedeaensis* (CW 17.2)	Lake Ossa	LZUY 15-3 (T351-30)	Mvogo Ndongo et al. 2017c	KY964474	KY964479	KY964472
*Bueamundemba*. (CW 26.2)	Korup N. P.	ZMB Crust. 30321	Mvogo Ndongo et al. 2019	MN188069	MN217388	MN217396
*Bueabangem* (CW 26.5)	Bakossi N.P.	IFAS-010	Mvogo Ndongo et al. 2020	MT019691	MT021447	–
*Bueaasylos* (CW 25.4)	Buea and Kumba	NHM 1994.588-591	Daniels et al. 2015	KP640489	KP640410	KP640453
*Potamonemusman* sp. nov	Bakossi N. P.	ZMB Crust. 30328	Mvogo Ndongo et al. 2019	MN188067	MN217390	MN217398
*Potamonemusman* sp. nov	Mt. Manengouba R.	ZMB Crust. 30320	Present study	GenBank (submitted by the first author)	–	–
*Potamonemusmambilorum*	Southwest Cameroon	NHM 1991.183	Daniels et al. 2015	–	KP640409	KP640452
*Potamonemussachsi*	Southwest Cameroon	NMU09.04.1983	Daniels et al. 2015	–	AY803490	AY803530
*Potamonautesidjiwiensis*	D. R. Congo	SAM A78437	Daniels et al. 2015	KP640481	KP640402	KP640446
*Potamonautesobesus*	Tanzania	Unaccessioned	Daniels et al. 2015	AY803647	AY803497	AY803537
*Afrithelphusamonodosa*	Guinea	NMU 25.IV.2005.C	Daniels et al. 2015	KP640469	KP640386	KP640430
*Globonautesmacropus*	Liberia	NMU VII. 1988	Daniels et al. 2015	–	KP640391	KP640435
*Sudanonautesaubryi*	Cameroon	LZUY-06	Mvogo Ndongo et al. 2017c	KY069938	KY964475	KY069950
*Sudanonautestiko*	Cameroon	ZMB Crust.29628	Mvogo Ndongo et al. 2017c	KY069941	KY964476	KY069954

LZUY: Zoological Collection of the Laboratory of Zoology, University of Yaounde 1, Cameroon; NHM: Natural History Museum, London, UK; NMU: Northern Michigan University Museum, USA; NP, National park; ZMB: Museum für Naturkunde, Berlin, Germany.

###### Diagnosis.

Carapace anterior surface smooth except for faint urogastric groove (Fig. 1d). Broad epimeral (longitudinal) suture on carapace sidewall (branchiostegite) dividing carapace sidewall into 2 regions, vertical (pleural) groove lacking (Fig. 1d). Outer lower margin of cheliped merus lined by small, blunt teeth, inner lower margin smooth, distal meral tooth distinct, pointed (Fig. 3d). Major chela dactylus straight (not arched) (Fig. 5g). Sternal sulcus S2/S3 completely traversing sternum; S3/S4 incomplete, reduced to 2 short, distinct notches on each side of sternum (Fig. 3d). G1 with long TA (TA/SS 0.66), slim, curving outward, lacking marginal setae, tapering to pointed tip; G2TA remarkably short (TA/SS 0.13) (Fig. 6j, l). A small species, mature between CWs 20–25 mm.

###### Description.

Carapace ovoid, medium height (CH/FW 1.17), wide (CW/FW 3.19); carapace surface smooth; postfrontal crest distinct, completely traversing carapace, lateral ends meeting anterolateral margins (Fig. 1d); exorbital tooth low, distinct; intermediate, epibranchial teeth each reduced to small granule (Fig. 4d); anterolateral margin behind epibranchial tooth smooth (Fig. 4d). Carapace branchiostegite with prominent epimeral suture dividing wall into subhepatic/suborbital, pterygostomial regions; vertical (pleural) suture faint (Fig. 3d). Sternal sulcus S2/S3 deep, completely traversing sternum; S3/S4 incomplete, reduced to 2 short, distinct notches on each side of sternum (Fig. 3d); margins of S3, S4 raised, broad (Fig. 3d); episternal sulci S4/E4, S5/E5, S6/E6 faint or missing, S7/E7 complete (Fig. 3d). Mandibular palp 2-segmented; medium-sized anterior lobe at junction between segment (0.25 × TS length; Fig. 7h). Third maxillipeds filling entire buccal cavern, except for transversely oval, efferent respiratory openings in superior lateral corners; ischium smooth, lacking vertical groove; exopod lacking flagellum (Fig. 7d).

Male chelipeds greatly unequal, right cheliped larger than left (Figs 1d, 5g, h). Movable finger (dactylus), fixed finger (pollex of propodus) of right (major) chela both slim, elongated; fixed finger with 3 large pointed teeth (2 proximal, 1 distal); movable finger relatively stout, straight (not highly arched), with 4 small but distinct teeth (2 proximal, 2 distal; Fig. 5g). Left (minor) chela dactylus, propodus smaller than right chela, with small teeth on occluding margins (Fig. 5h). Inner inferior margin of cheliped merus lined by small teeth, outer inferior margin smooth; distal meral tooth large, pointed (Fig. 5d). Cheliped carpus inner margin with large pointed distal tooth; proximal tooth much smaller, followed by granule (Fig. 5l). Ambulatory legs (P2–5) slender, P4 longest, P5 shortest; dactyli P2–5 tapering to point, each bearing rows of downward-pointing sharp bristles, P5 dactylus shortest (Fig. 1d).

Male pleon triangular, margins not indented and lacking setae (Fig. 3d). G1 with long TA (TA/SS 0.66), slim, curving outward, lacking marginal setae, tapering to pointed tip; G2TA remarkably short (TA/SS 0.13) (Fig. 6j, l); G1SS, broad in basal, midsection, distal quarter tapering sharply, narrow at junction with G1TA (Fig. 6j, l). G2TA extremely short (G2TA/SS 0.3; Fig. 6k).

***Adult female.*** Right and left chelipeds subequal. Fixed, movable fingers of chela interspersed with series of smaller acute teeth along their length. Pleon wide, covering entire sternum, reaching bases of coxae of P2–5; pleon with 6 free pleomeres (A1–6) becoming gradually wider proximally, telson wide, forming near semicircle.

***Size.*** Small species, CW in mature individuals ranging from 20.0–30.4 mm.

***Colour in life.*** Dorsal carapace and all ambulatory legs dark brown, chelipeds red.

###### Type locality.

Stream flowing into Man Lake, Mount Manengouba, in Manengouba Ecological Reserve, Southwest Region of Cameroon.

###### Etymology.

The species is named for Man Lake, one of a pair of small lakes in the caldera at the summit of Mount Manengouba (the other lake being Woman Lake). The species epithet is used as a noun in apposition.

###### Habitat.

At the Man Lake locality at the summit of Mount Manengouba the species is found in a small stream flowing into the lake, and it was also collected from a small stream in the Bakossi National Park. Both of these localities are located in rainforest habitat found along the Cameroon Volcanic Line, a 1,600 km long chain of volcanoes that stretches from the islands in the Gulf of Guinea to the mountains of eastern Nigeria and western Cameroon, including Mount Cameroon.

###### Remarks.

The new species is assigned to *Potamonemus* because it conforms to the genus diagnosis (Cumberlidge and Clark 1992; Cumberlidge 1993c; Cumberlidge et al. 2019). *Potamonemusman* sp. nov. most closely resembles *P.sachsi* in that the dactylus of the major cheliped of both species is straight rather than highly arched. These two species can be distinguished from each other by the smooth carapace and branchiostegal sidewalls in *P.man* sp. nov. (Figs 1D, 2D) (vs. patches of short setae along the anterolateral and posterolateral margins of the carapace that continue around to the sidewalls in the subhepatic and pterygostomial regions of the branchiostegite in *P.sachsi* (Cumberlidge et al. 2019: fig. 4c)). The highly arched dactylus of the major cheliped of *P.mambilorum* distinguishes it from both *P.man* sp. nov. and *P.sachsi*. Finally, *P.man* sp. nov. can be distinguished from *P.mambilorum* and *P.sachsi* by the body size of adult specimens: the new species and *P.sachsi* are adult between CW 20–30 mm and CW 23–28 mm, respectively, while *P.mambilorum* is the largest species (adult at CW 29–38 mm).

**Figure 8.**
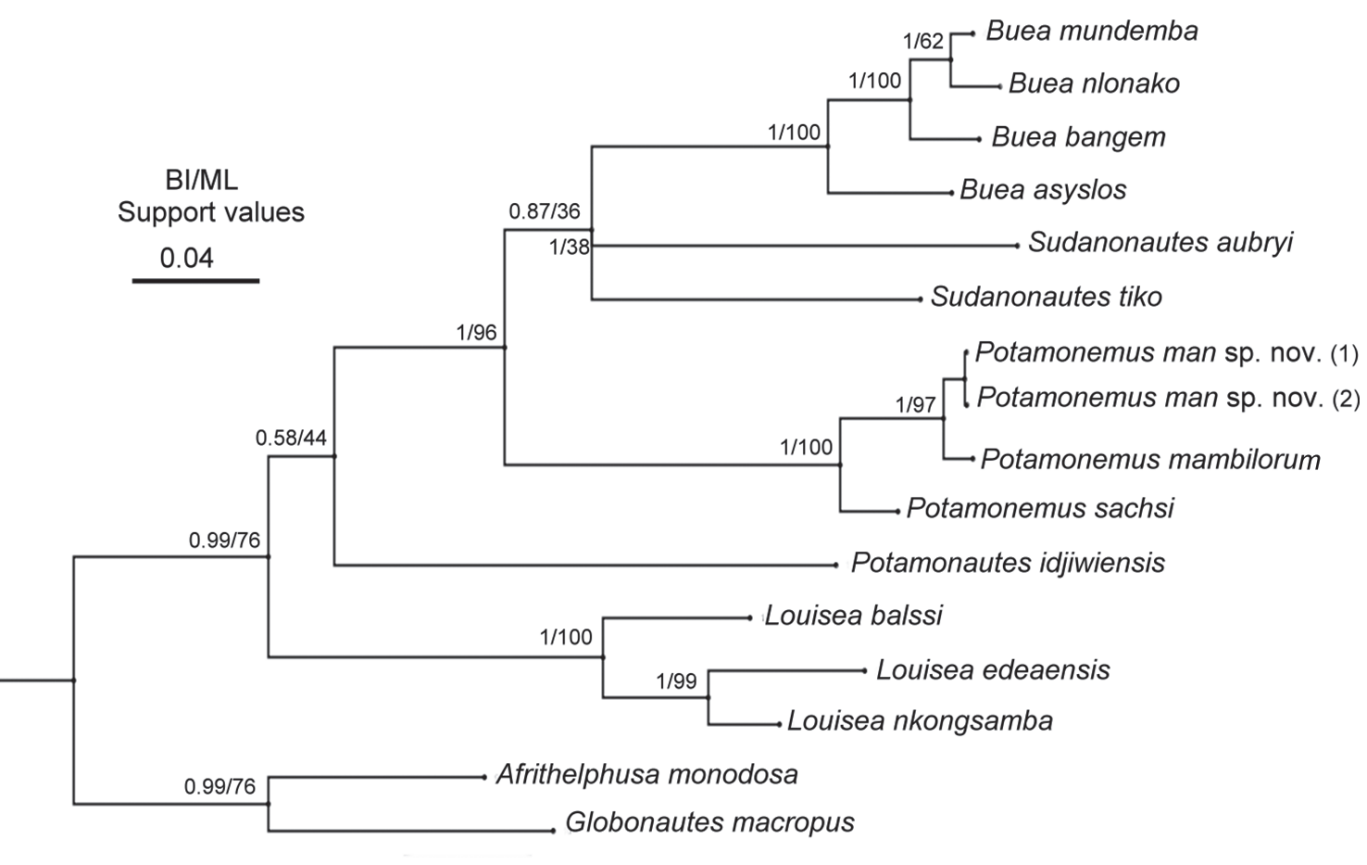
Bayesian Inference (BI) tree topology for the freshwater crab taxa from Cameroon included in this study derived from mtDNA sequences corresponding to three loci (partial 16S rRNA, COI, and 12S rRNA genes). Bayesian Inference (BI) and ML statistical values (%) on the nodes indicate bootstrap support and posterior probabilities, respectively.

A phylogenetic tree (Fig. 8), based on 1,848 base pairs representing the combined partial sequences of three mtDNA markers (COI, 16S RNA, and 12S RNA), recovered three species of *Potamonemus* as a single clade with strong BI and ML confidence values (1/100 at this node). The three species of *Potamonemus* (*P.mambilorum*, *P.man* sp. nov., and *P.sachsi*) form an independent lineage within the subfamily Potamonautinae, and all are found in the same geographical area of Cameroon. The uncorrected *p*-distance between *Potamonemusman* sp. nov. and *P.mambilorum* is 2.2% for 12S RNA and 0.6% for 16S RNA, and between *P.man* sp. nov. and *P.sachsi* it is 4.5% for 12S RNA and 4.6% for 16S RNA.

###### Conservation.

*Potamonemusman* sp. nov. is found in an area of great conservation interest. The aquatic habitats of this species in the Mount Manengouba Ecological Reserve and in the Bakossi National Park are both in montane tropical rainforest in the Cameroon highlands, an area with a high biodiversity and a high rate of endemism, including freshwater crabs (Cumberlidge et al. 2019; Mvogo Ndongo et al. 2017a, d, c, 2018, 2019, 2020). In Mount Manengouba Ecological Reserve the small, forested stream near Man Lake where *P.man* sp. nov. lives also supports a sympatric population of the endangered freshwater crab *Louiseabalssi* (Bott, 1959). Significantly, there are no reports of any species of invertebrates (molluscs, insects, crustaceans) or vertebrates (fish, amphibians, snakes, and birds) from Man Lake itself. This inhospitality to life may be related to the unusual green colour of its waters which may be due to the accumulation of lethal compounds, which may also be a potential danger to humans (see Mvogo Ndongo et al. 2018). Both the Mount Manengouba Ecological Reserve and the Bakossi National Park are under increasing pressure from growing nearby human populations and from the associated clearance of land for agriculture. As a result, despite being found in protected areas, the habitat of *P.man* sp. nov. is increasingly threatened by nearby intensive agricultural practices and forest destruction for firewood collection. In addition, the farmers encroaching on these habitats use agrochemicals and pesticides on their crops, and these pollutants eventually drain into the aquatic systems, potentially poisoning the freshwater communities (Mvogo Ndongo et al. 2018).

##### Potamonemus mambilorum

Taxon classificationAnimaliaDecapodaPotamonautidae

Cumberlidge & Clark, 1992

F7A19E54-7650-576E-997F-BB44B56700A0

Figures 1b , 2b , 3b , 4b , 5c, d, i , 6d–f , 7b, f

###### Material examined.

6 adult ♂ (CW 29.05 mm, CL 21.17 mm, CH 12.10 mm, FW 8.85 mm; CW 29.56 mm, CL 21.35 mm, CH 12.34 mm, FW 9.12 mm; CW 29.16 mm, CL 20.70 mm, CH 12.00 mm, FW 9.12 mm; CW 28.93 mm, CL 20.69 mm, CH 11.85 mm, FW 9.94 mm; CW 26.74 mm, CL 19.62 mm, CH 11.32 mm, FW 9.63 mm; CW 26.74 mm, CL 19.62 mm, CH 11.32 mm, FW 9.63 mm). 2 adult ♀ (CW 27.06 mm, CL 19.76 mm, CH 12.45 mm, FW 8.34 mm; CW 26.68 mm, CL 19.06 mm, CH 11.03 mm, FW 7.72 mm); Cameroon, Littoral region, Mount Nlonako Ecological Reserve (4.891820, 9.984830), 900 m a.s.l., 26 May 2018, coll. P.A. Mvogo Ndongo (ZMB Crust. 32428). 1 adult ♂ (CW 28.00 mm, CL 19.10 mm, CH 11.37 mm, FW 8.56 mm); 1 adult ♀ (CW 28.36 mm, CL 20.00 mm, CH 10.27 mm, FW 7.79 mm; Southwest Region, Mount Manengouba Ecological Reserve, small stream around the mountain (ZMB Crust. 30326) (5.034920, 9.836150), 1,958 m asl, 14 March 2017, coll. P.A. Mvogo Ndongo.

###### Remarks.

The distributional range of *P.mambilorum* is extended in this work by the discovery of populations in Mount Nlonako Ecological Reserve in the littoral region of Cameroon. *Potamonemusmambilorum* was previously known from seven localities in the forested highlands and lowlands of southwestern Cameroon (extent of occurrence (EOO) 43,291 km^2^). The conservation status of this species was assessed as Least Concern (Cumberlidge 2008a), but this was before the threats to the freshwater ecosystems of this part of Africa were brought to light. The conservation status of *P.sachsi* was assessed as Vulnerable, B1ab(iii)+2ab(iii), based on its distributional range that includes the Bamenda highlands in southwest Cameroon and the neighboring Obudu plateau in southeast Nigeria, which is continuous with the Bamenda highlands (EOO 24,219 km^2^) and perceived threats (Cumberlidge 2008b). The areas where *P.mambilorum* and *P.sachsi* occur are now known to be at risk from a number of anthropogenic threats, including deforestation, together with intensive and encroaching agricultural practices and firewood collection, as well as release of pollutants such as agrochemicals potentially affecting the eggs, hatchling-carrying female crabs, and other aquatic organisms.

### Key to the species of *Potamonemus* Cumberlidge & Clark, 1992

**Table:** 

1	Dactylus of major cheliped highly arched (Fig. 5a)	***P.mambilorum***
–	Dactylus of major cheliped either straight or only slightly concave	**2**
2	Carapace sidewalls in subhepatic and pterygostomial regions smooth	***P.man* sp. nov.**
–	Carapace sidewalls in subhepatic and pterygostomial regions with fields of short setae (Cumberlidge 1994: figs 3a, 4c)	***P.sachsi***

## Supplementary Material

XML Treatment for Potamonemus man

XML Treatment for Potamonemus mambilorum
